# Evaluation of Feature Selection Methods for Classification of Epileptic Seizure EEG Signals

**DOI:** 10.3390/s22083066

**Published:** 2022-04-16

**Authors:** Sergio E. Sánchez-Hernández, Ricardo A. Salido-Ruiz, Sulema Torres-Ramos, Israel Román-Godínez

**Affiliations:** Division of Cyber-Human Interaction Technologies, University of Guadalajara (UdG), Guadalajara 44100, Jalisco, Mexico; sergio.sanchez1153@alumnos.udg.mx (S.E.S.-H.); ricardo.salido@academicos.udg.mx (R.A.S.-R.); sulema.torres@academicos.udg.mx (S.T.-R.)

**Keywords:** EEG, epilepsy, seizure detection, machine learning, features, feature selection

## Abstract

Epilepsy is a disease that decreases the quality of life of patients; it is also among the most common neurological diseases. Several studies have approached the classification and prediction of seizures by using electroencephalographic data and machine learning techniques. A large diversity of features has been extracted from electroencephalograms to perform classification tasks; therefore, it is important to use feature selection methods to select those that leverage pattern recognition. In this study, the performance of a set of feature selection methods was compared across different classification models; the classification task consisted of the detection of ictal activity from the CHB-MIT and Siena Scalp EEG databases. The comparison was implemented for different feature sets and the number of features. Furthermore, the similarity between selected feature subsets across classification models was evaluated. The best F1-score (0.90) was reported by the K-nearest neighbor along with the CHB-MIT dataset. Results showed that none of the feature selection methods clearly outperformed the rest of the methods, as the performance was notably affected by the classifier, dataset, and feature set. Two of the combinations (classifier/feature selection method) reporting the best results were K-nearest neighbor/support vector machine and random forest/embedded random forest.

## 1. Introduction

Epilepsy is one of the most common neurological diseases, affecting around 50 million people of all ages globally [1]. The Center for Surveillance, Epidemiology and Laboratory Services of the United States of America estimated that in 2010, the number of adults with active epilepsy in the United States was 2.3 million; by 2015, the estimate increased to 3 million adults [2]. An additional study calculated that the cumulative incidence of epilepsy, in Norwegian children at the age of ten was around 0.66%, with 0.62% having active epilepsy [3]. The authors in [4] reported six studies of epilepsy prevalence in Mexico, which found prevalence rates of 3.9 to 42.2 per 1000 inhabitants.

An important tool for the diagnosis and management of epilepsy is the electroencephalogram (EEG). As mentioned in [5] (p. ii2), EEG is a “convenient and relatively inexpensive way to demonstrate the physiological manifestations of abnormal cortical excitability that underlie epilepsy.” Other diagnostic techniques used in conjunction with EEG include neuroimaging, metabolic tests, and genetic tests. EEG can be processed and classified by using machine learning methods. Several studies have applied machine learning to classify ictal EEG signals [6,7] and predict seizures [8,9]. Concerning signal description, several metrics have been used to characterize such problems, and to build machine learning models, some of those metrics are computed from an EEG. Some researchers have approached the classification problem by calculating statistical, entropy, and univariate linear metrics from EEG [6,8,9,10,11]; those metrics can be fed to a model in the form of a vector or a matrix. In addition, metrics can be estimated for the entire EEG bandwidth or for smaller sub-bands [12,13,14], the latter with the intention of obtaining a more detailed view of the signal. Furthermore, transformation of the EEG signal to different domains has been explored by applying Fourier [15], short-term Fourier [7,16,17], wavelet transform [15,16,18,19,20], and contourlet transform [21]. It has also been analyzed as a graph [8] and an image [16]. As a result of the increasing interest in this topic, Ref. [22] presented a complete summary of several descriptors for time-domain, frequency-domain, and time–frequency-domain, along with their interpretation, while applying to the epileptic seizure detection on EEG signals.

Regarding the classification problem, a diversity of machine learning models have been tested for epilepsy prediction; among these we have: decision trees [23,24,25], support vector machine [23,26,27], K-nearest neighbor [23], and random forest [27]. In recent years, diverse deep learning models have been tried for epilepsy-related tasks, e.g., convolutional neural networks [16,17,28] and long-short term memory [8,28].

An important step in classifier modeling is feature selection. When performing feature selection, it is important to take into account several facts: (a) there are numerous attributes that can be calculated from EEG signals—each one describing a particular aspect of EEG; (b) there is a strong relation between features and model accuracy; (c) the curse of dimensionality, which is related to the difficulty of optimizing a solution in high-dimensional spaces; (d) the complexity and interpretability of the resulting models, which are about reducing the time and costs by training simpler learning models or selecting the features that are more relevant and meaningful from the problem perspective [29]. In this sense, several feature selection methods have been used to overcome the aforementioned issues. Some of those techniques are statistical tests, information gain [30], principal component analysis [31], permutation importance [32], and recursive feature elimination (RFE) [33], among others [29].

In this regard, there are several works in the state-of-the-art focused on epileptic EEG signal classification; some of them use feature selection methods (FSMs) to improve their results and reduce the dimension of the feature vectors.

For example, principal component analysis was applied in [34] to obtain less correlated features; however, their main goal was to evaluate the effects of channel selection on epileptic analysis over adults and children, without considering the effect of the feature selection method in the classification’s performance, and only one classification method was considered, in this case linear discriminant analysis.

In [24], RFE was used to rank features and a support vector machine (SVM) for classifying epilepsy, autism, and control groups in children. Even though they determined which features and combinations of features contributed the most to the classification accuracy, they did not analyze either several feature selection methods or other classification methods.

The authors of [25] evaluated one feature selection method (recursive feature elimination) and one feature set, in combination with seven classifiers to improve the classification accuracy of automatic seizure diagnosis. From the 11 features that were calculated from the EEG signal, they reduced them to 8 features. All the experiments were performed only on one adult dataset.

In [12], six FSMs along with nine classifiers were used for automatic seizure detection. The FSMs were evaluated to rank and reduce the number of features, ranking the important features using a *t*-test and selecting the top 20 or 25, without testing additional alternatives that may result in different rankings. Furthermore, the authors provided experimental results based on one dataset that belonged to children. Additionally, the hold-out cross-validation methodology was used, which is commonly used for bigger datasets.

FSMs have also been tested in signals other than EEG such as magnetic resonance images, as shown in [35]. The authors’ objective was to compare three different FSMs (i.e., *t*-test filtering, the sparse-constrained dimensionality reduction model, and the support vector machine-recursive feature elimination) to determine which of them performed better when using an SVM as the classifier. However, the authors only tested the performance of the FSM on one classifier (SVM). Furthermore, they only tested on one dataset without considering if their result may change when either tested on different datasets or FSM sets.

In summary, it can be observed that, even though there is extensive research about the feature selection methods applied in seizure and seizure-free EEG signals’ classification, such approximations do not allow having a general perspective of the advantages and disadvantages of using a particular combination of the feature selection method with a classification algorithm (C-FSM) to determine: (1) the effect of the dimensionality reduction on the performance of the classifiers; (2) the best C-FSM combinations; and (3) the amount of coincidence of the best-selected features among FSMs; all of that considering two different feature sets and two different databases (adults and children).

Hence, in this work, the CHB-MIT and Siena Scalp EEG databases were used along with two different feature sets to evaluate the combination of six FSMs and five classification models. The results of this work allow determining: the minimum number of features that can be chosen for each FSM without scarifying the classifiers’ performances; the performance of several C-FSM combinations in order to discover if a relationship exists between the FSMs with a particular classification algorithm; and if there is a feature or feature set that remains across different C-FSM combinations.

## 2. Materials and Methods

**Figure 1:**
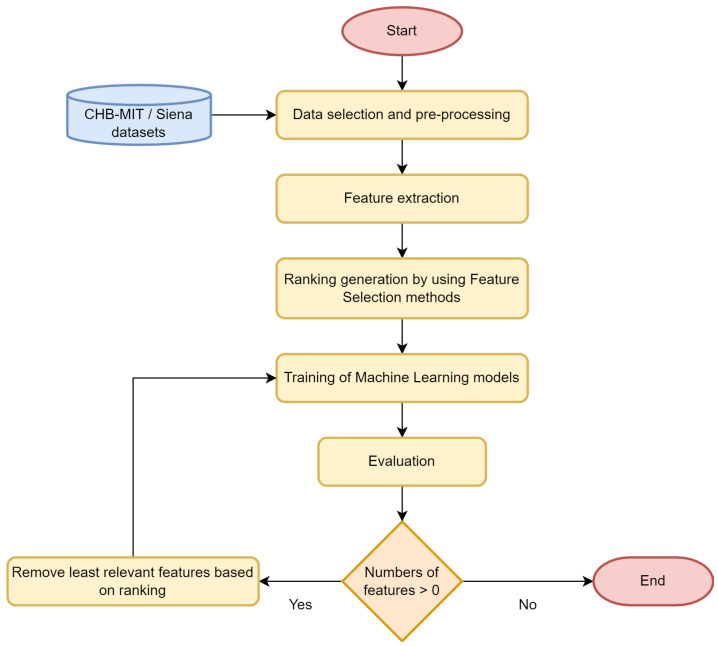
Flow diagram of the methodology.

In this section, the features, models, and training procedures are described. A general methodology overview is shown in Figure 1.

### 2.1. Datasets

CHB-MIT was one of the two datasets used for this research. The dataset is available for download at Physionet [36] under Open Data Commons Attribution License v1.0.

The data were collected at the Boston Children’s Hospital. The database contains scalp electroencephalograms of 23 pediatric patients having epilepsy [37]. The number of recordings per patient varies from 9 to 45; all of them contain a metadata file listing the channels’ names and ictal activity intervals; most of the records have a duration of one hour. EEG signals were sampled at 256 Hz; electrodes were placed according to the 10–20 system [37]. Most of the recordings are provided following a bipolar longitudinal montage.

After exploring the data, recordings not containing ictal activity were discarded. Thus, an amount of 137 EEG recordings containing an overall amount of 181 ictal activity intervals from 23 patients were accomplished.

The second dataset used in this research was the Siena Scalp EEG database; it is also available on the Physionet site under a Creative Commons Attribution 4.0 license. The dataset was collected by the Unit of Neurology and Neurophysiology at the University of Siena. It contains EEG recordings of 14 patients, 9 males (ages 36–71) and 5 females (ages 20–58). There are a total of 41 EEG recordings, and these include 47 ictal activity intervals; the recordings’ duration is variable, from 1 to 13 h. The start and end of each seizure are detailed in the metadata file provided by the original authors [38]. EEG signals were sampled at 512 Hz. Electrodes were placed according to the 10–20 system [38]. EEG channels provided in the dataset are monopolar.

Recordings were further processed to design a dataset for a bi-class classification problem: seizure or seizure-free.

### 2.2. Data Pre-Processing

The number of conserved bipolar channels for the CHB-MIT recordings was 21. A few of the channels do not follow the 10–20 positioning, but channels were considered because these are included in every recording of the dataset. The Siena Scalp recordings were converted to a longitudinal bipolar montage, to have a fair comparison between both datasets; 18 channels were conserved.

A second-order Butterworth high-pass filter was used for removing frequencies below 0.5 Hz; then, a notch filter was applied to remove power line frequency (60 Hz and 50 Hz for CHB-MIT and Siena datasets, respectively).

A window length of 2 s was chosen. Epoch length was based on [13], as they mentioned, when extracting spectral features, it is important to choose small epochs due to the non-stationarity of the EEG.

As the number of ictal segments was significantly lower than the non-ictal ones, a 50% overlap was applied to ictal windows; besides that, some non-ictal windows were removed in order to keep an approximate ratio of 9:1 between both classes.

Ten percent of the CHB-MIT epochs were kept apart for adjusting the parameters of the classification models.

Inspired by [12], windowed EEG signals were separated into the following 5 sub-bands: complete bandwidth (0.5–30 Hz), delta (0.5–4 Hz), theta (4–8 Hz), alpha (8–12 Hz), and beta (12–25 Hz). Second-order Butterworth band-pass filters were used for band separation.

### 2.3. Feature Extraction

Two different features sets were evaluated to observe the effect of varying the metrics and determine which one outperforms (see Table 1). Therefore, half of Feature Set 1 (FS1) was conformed by statistical metrics and was applied in the time-domain or frequency-domain. Specifically, the median frequency (i) was estimated to characterize the power spectrum of the EEG data. On the other hand, the variance (ii), skewness (iii), and kurtosis (iv) were estimated in time-domain to characterize the variability and the distribution of the EEG data. Other features such as the peak frequency (v) were used to describe the frequency of the highest peak in the power spectral density; the root mean square (vi), range (vii), and the number of zero crossings (viii) in the time-domain were used respectively to estimate the effective value of the signal, to measure the maximum wave amplitude, and to count the number of points where the EEG wave cuts the horizontal axis, changing its state from positive to negative and vice versa.

Those features were selected based on previous studies where the authors performed an extensive review on the state-of-the-art on epileptic seizure detection based on EEG [22] along with some other works [6,8,10,12,25,34] that have successfully applied such feature metrics. In particular, Features i–vi were selected based on [12]. The number of zero crossings (ZCs) was estimated instead of the ZC rate because all EEG epochs had the same length.

On the other hand, FS2 was formed by the minimum (ix), which describes the minimum value that a signal can take, complexity (x), used to describe the change in frequency, mobility (xi), which is a measure of the mean frequency, interquartile range (xii), which is a measure of statistical dispersion, the spread of the data or observations, median absolute deviation (xiii), which is a robust measure of how spread out a set of data is, sample entropy (xiv), used for assessing the complexity of physiological time series signals, mean (xv), and standard deviation (xvi), statistical parameters that describe the average value and the amount of variability, or dispersion, from the individual data values to this average value. These features have been utilized across different studies for the classification of ictal EEG data [6,10,11,12,34]. Parameters used for the sample entropy (SampEn) are r = 0.2, m = 2. When applied to seizure discrimination in EEG, it was previously observed [44] that there is no best parameter combination, but several optimal combinations, one of which is the one used in this work.

The features from FS1 and FS2 were computed per channel and frequency band. For both feature sets (FS1 and FS2), feature vectors of 840 and 720 in length were obtained for the CHB-MIT and Siena datasets, respectively.

After that, correlations between features in all bands and channels were identified in both feature sets. For each pair of features, the Pearson correlation coefficient was computed. If a combination had a coefficient over 0.95, one of the features of the pair would be discarded. As a result, from FS1, 105 and 113 features were removed for the CHB-MIT and Siena datasets, respectively; on the other hand, from FS2, 304 and 257 features were removed for the CHB-MIT and Siena datasets, respectively.

### 2.4. Classification Methods

The following five algorithms were selected for classifying ictal EEG signals:

1. Decision tree (DT): This is a hierarchical model composed of decision nodes and terminal leaves, each leaf have an output label. Decision nodes implement a test function f(x), which is a discriminator dividing the input space into smaller regions. Among all possible splits, the DT looks for the one that minimizes impurity. There are several impurity measures, e.g., the Gini index and entropy. For a two-class problem, the Gini index is defined as [45]:
  (1) ϕ(p,1−p)=2p(1−p)
  where *p* is the probability of a sample reaching a node *m*, to belong to a class *C*. The classification and regression trees algorithm (CART) was applied in this research.
2. Support vector machine (SVM): This constructs a hyperplane or set of hyperplanes in a high-dimensional space that can be used for classification. Those hyperplanes have the largest distance to the nearest training data points (also known as the functional margin) [23]. The task of finding the optimal separating hyperplanes can be defined as [45]:
  (2) $$min\frac{1}{2}{\left||w|\right|}^{2}\;\mathrm{subject}\;\mathrm{to}\;r^{t}\left(w^{T}x^{t}+w_{0}\right)\geq 1,\forall t$$
  where *w* are the parameters defining the hyperplane, $x^{t}$ are the instances of the training set, and $r^{t}$ is the actual label. If the problem is not linearly separable, the problem can be mapped to a new space by using non-linear basis functions [45].
3. K-nearest neighbor (KNN): This is a classifier that learns by analogy. A target unknown instance is compared to all the instances in the training set, locating the *k* closest instances; the algorithm assigns the class that corresponds to the majority. “Closeness” is measured by using a distance metric; in this study, we used the Manhattan distance (selected through a parameter grid search). The Manhattan distance for a *p*-dimension space is defined as [46]:
  (3) $$d\left(i,j\right)=|x_{i1}-x_{j1}\left|+\right|x_{i2}-x_{j2}\left|+\ldots +\right|x_{ip}-x_{jp}|$$
4. Random forest (RF): This is an ensemble method, and each classifier of the ensemble is a DT. For each node of the DT, a random selection of features is used to determine the best split. RF also uses bagging (bootstrap aggregation), which means that the training set for each DT is sampled with replacement from the original training set. After model training, each DT votes, and the most voted class is assigned to the test instance [46].
5. Artificial neural network (ANN): This is a classifier that uses the idea of the perceptron, and it is referred to as a multi-layer perceptron. It can contain three or more layers, and these are: an input layer, one or more hidden layers, and an output layer [19]. Simply speaking, it is a set of input/output interconnected units, each connection having a weight associated with it. During the learning process, the ANN adjusts the weights of the connections in order to be able to predict the correct labels of new instances [46]. The neural network used in this study is described in Table 2.

To select the best-suited parameters for the above machine learning models, a grid search was performed; accuracy was utilized as a comparison metric. For selecting the ANN structure, a different number of neurons and hidden layers (1 and 2 layers) were evaluated, resulting in our best architecture, the one depicted in Table 2. The configuration settings and parameters of the grid search are listed in Table 3 for FS1 and in Table 4 for FS2. Both FSs used the same parameter grid detailed in Table 3. It is worth mentioning that the selected classification algorithms have been previously implemented for epilepsy-related tasks; however, the model parameters were not inspired by any specific work; on the contrary, they were selected by performing a grid search.

### 2.5. Feature Selection Methods

In this research, six FSMs were used. The metrics used to assign the importance of each feature are detailed below. The parameters selected for training each algorithm are mentioned in Table 5.

1. Decision tree (DT): The measure used to assign the feature importance is the Gini importance. As described in [47], the importance of feature $X_{m}$ in an RF can be measured by Equation (4)
  (4) $$Imp\left(X_{m}\right)=\frac{1}{N_{T}}\sum_{T}\sum_{t\in T:v\left(s_{t}\right)=X_{m}}p\left(t\right)▵i\left(s_{t},t\right)$$
  where *T* is a set of DTs, $v(s_{t})$ is the feature used to split node *t*, $▵i(s_{t},t)$ is the impurity decrease in node *t*, and p(t) is:
  (5) $$p\left(t\right)=N_{t}/N$$
  *N* is the number of training samples, and $N_{t}$ is the number of samples reaching the node *t*. As this study uses a single DT, the size of *T* is 1.
2. Support vector machine (SVM): Coefficients estimated by SVM can be utilized to produce features’ ranking [33]. A linear SVM is trained on the input dataset, then features are ranked as per the absolute values of the hyperplane weights [48].
3. Local interpretable model-agnostic explanations (LIME): This is a technique for explaining the predictions of any classifier by learning an interpretable model locally around the prediction. For a classification model *f* and interpretable model class *G*, the explanation is obtained by optimizing Equation (6) [49].
  (6) $$\xi \left(x\right)=\arg\min_{g\in G}L\left(f,g,\pi_{x}\right)+\Omega \left(g\right)$$
  Function *L* measures the approximation of *g* to *f* in the locality defined by $\pi_{x}$. Function Ω(g) is a measure of the complexity of *g*. As per [49], the exponential kernel was used for $\pi_{x}$, the weighted square loss for *L*, and the linear model for *G*.
4. Shapley additive explanations (SHAP): This is a unified approach to interpreting model predictions. It assigns each feature an importance value or SHAP value. These are the Shapley values of a conditional expectation function of the original model [50]. The implementation applied in this study, kernel SHAP, optimizes (6), but it uses different forms of $\pi_{x^{\prime }}$, *L*, and Ω:
  (7) Ω(g)=0
  (8) $$\pi_{x^{\prime }}\left(z^{\prime }\right)=\frac{M-1}{\left(M\;choose\;\right|z^{\prime }\left|)\right|z^{\prime }\left|(M-\right|z^{\prime }\left|\right)}$$
  (9) $$L\left(f,g,\pi_{x}^{\prime }\right)=\sum_{z^{\prime }\in Z}{\left[f\left(h_{x}\left(z^{\prime }\right)\right)-g\left(z^{\prime }\right)\right]}^{2}\pi_{x^{\prime }}\left(z^{\prime }\right)$$
  where *g* is the explanation model and follows a linear form, *f* is the classification model, *M* is the total number of features, and $|z^{\prime }|$ is the number of used features. Parameter $z^{\prime }$ is the set of features represented as ${\left\{0,1\right\}}^{M}$. As explained in [51], the function $h_{x}$ maps the 1s of $z^{\prime }$ to the value from the instance to be explained *x*, and 0s are replaced by a random feature value of another instance sampled from the data.
5. Embedded random forest (ERF): “feature importance is measured by randomly permuting the feature in the out-of-bag samples and calculating the percent increase in misclassification rate as compared to the out-of-bag rate with all variables intact” [48] (p. 319). This technique was originally described in [52].
6. Reciprocal ranking (RR): Known also as inverse rank position [53], this is an ensemble method that merges ranked lists into a single one *r* based on Equation (10) [54]:
  (10) $$r\left(f\right)\;=\;\frac{1}{\sum_{j}\frac{1}{r_{j}\left(f\right)}}$$
  where $r_{j}\left(f\right)$ is the ranking position assigned to a feature by the rest of the FSMs.

Instances of the SHAP background dataset (Table 5) were estimated by applying K-means (k=50) to a subset of the training dataset. This was done in order to use a small, but representative set of instances for the estimation of the SHAP values. For feature evaluation in ERF, first, RF was trained, then permutation importance was estimated in a separate dataset.

The DT and SVM were chosen due to simplicity and training speed. SHAP, ERF, and RR were selected because, in [54], these methods returned good performance and consistency for a prediction task related to environmental datasets. LIME was considered because it is model-agnostic and allows model interpretation, similar to SHAP.

### 2.6. Model Training and Evaluation

Feature importance rankings were obtained for each combination of feature selection and classification method, and as a result, 30 feature rankings were estimated per dataset. For convenience, if any negative importance score was assigned to a feature, the absolute value was calculated. As LIME and SHAP compute feature importance per instance, the average was computed across all instances. When model training was required during ranking computation, 50% of the training dataset was passed to the model, and min–max normalization [46] was used for data scaling.

During the training and evaluation of the classification models, data were min–max-normalized and a 5 × 2 cross-validation (2-fold, 5 repetitions) was implemented for model evaluation [55]. On each database (i.e., CHB-MIT and Siena), patients’ epochs were merged into a single dataset; then, each new dataset was randomized after each iteration of the validation methodology.

The 5 × 2 CV F-test procedure was originally proposed to compare supervised classification algorithms, even though it has been previously implemented for comparison of FSMs (not applied to EEG data) [56,57].

First, the classification models were trained by keeping all the features in the training set and assessed in order to compute their classification performance. Then, features having the smallest ranking criterion were removed, and the model was re-trained and re-evaluated. Feature removal was performed in steps of 50 features at a time. There were 25, 12, 6, and 1 features also considered during the evaluation. Rankings were computed at the beginning of the process.

It should be noted that the above pipeline was repeated 6 times per classification method, as there were 6 FSMs. In addition, models were trained per each dataset separately.

### 2.7. Computing and Software

The experiments were run on 2 different computing devices: a computer with Intel Core i7, 12 GB of RAM, and Ubuntu 18.04 and a server with Intel Xeon Gold and NVIDIA Tesla P100. Python 3.7 was used for coding all the experiments. Numpy (1.19.5) [58], pandas (1.2.2) [59], and scipy (1.6.1) [60] were used for data engineering, scikit-learn (0.24.1) [61] and tensorflow (2.4.1) [62] for building machine learning models and feature selection algorithms, and matplotlib (3.3.4) [63] for plotting. Other needed libraries were lime (0.2.0.1) [49] and shap (0.39.0) [50]. Some processing pipelines were run on a Jupyter Notebook in order to visualize the charts.

## 3. Results

### 3.1. Evaluation of Feature Dimensionality Reduction

This analysis was performed in order to visualize the effect of the reduction of the feature vector size on the classification models’ performance. By doing this, it was possible to have a general overview of the robustness of the classification models regarding the reducing of the feature vectors that may result from feature selection methods.

**Figure 2:**
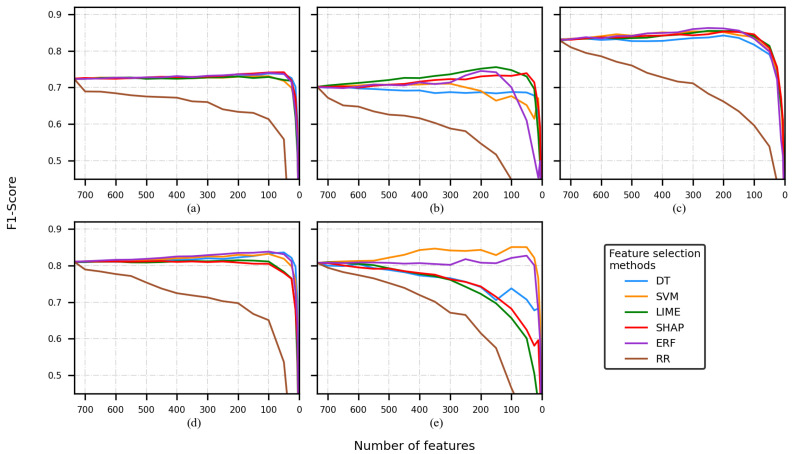
F1-score of the model was obtained by using a different number of features, using the CHB-MIT dataset and FS1. Classification model used: (**a**) decision tree, (**b**) support vector machine, (**c**) artificial neural network, (**d**) random forest, and (**e**) K-nearest neighbor.

**Figure 3:**
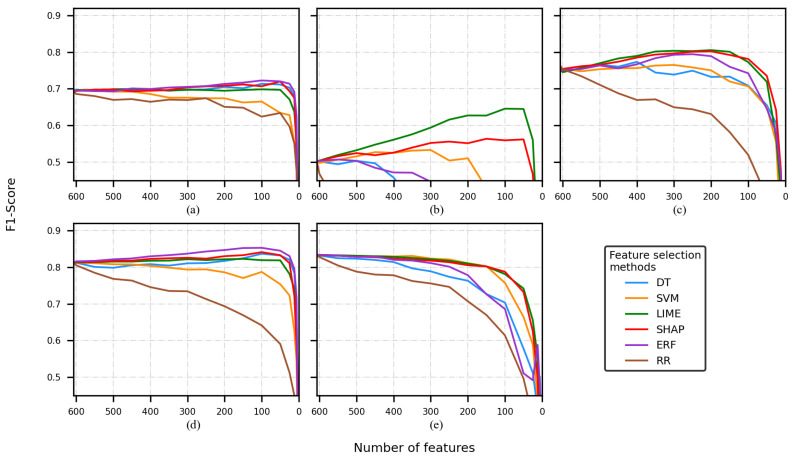
F1-score of the model is obtained by using a different number of features, using the Siena dataset and FS1. Classification model used: (**a**) decision tree, (**b**) support vector machine, (**c**) artificial neural network, (**d**) random forest, and (**e**) K-nearest neighbor.

**Figure 4:**
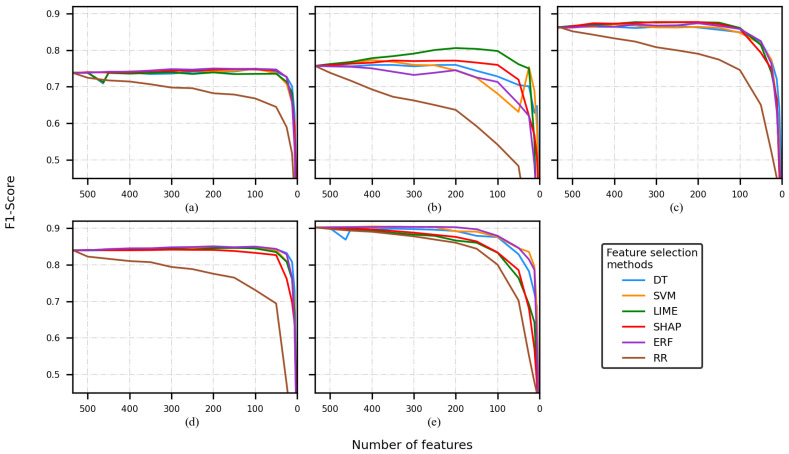
F1-score of the model is obtained by using a different number of features, using the CHB-MIT dataset and FS2. Classification model used: (**a**) decision tree, (**b**) support vector machine, (**c**) artificial neural network, (**d**) random forest, and (**e**) K-nearest neighbor.

**Figure 5:**
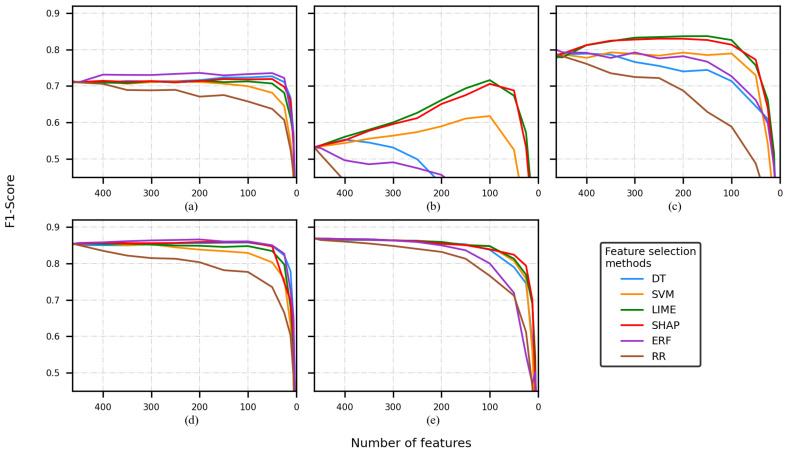
F1-score of the model is obtained by using a different number of features, using the Siena dataset and FS2. Classification model used: (**a**) decision tree, (**b**) support vector machine, (**c**) artificial neural network, (**d**) random forest, and (**e**) K-nearest neighbor.

Figure 2, Figure 3, Figure 4 and Figure 5 present five plots depicting the average F1-score for the combination classifier (C), feature selection method (FSM), and a number of features (NF) (C-FSM-NF). Every subplot corresponds to a different classification model, namely (a) decision tree (DT), (b) support vector machine (SVM), (c) artificial neural network (ANN), (d) random forest (RF), and (e) K-nearest neighbor (KNN). Colored lines indicate the average performance for different NF values, and each color indicates a different FSM.

When the CHB-MIT dataset and the FS1 were used, the best performances were reported by the ANN model and the worsts by the DT and SVM models. The former reported the best F1-score with 0.86 corresponding to the combination ANN-ERF-250/200 (see Figure 2c). Additionally, it was observed that for every classifier, the RR feature selection method (brown line) decreased from the early stages (see Figure 2).

The second-best-performing classifier was RF (see Figure 2d); most of the F1-score values were between 0.80 and 0.85. Most of the curves showed similar tendencies, but the RR curve (brown line) went down faster, again.

KNN (see Figure 2e) returned few F1-scores that overcame the RF classifier, but it was less stable (i.e., its performance was more dependent on the FSM). A pair of the curves was over 0.80 (yellow and purple lines corresponding to the SVM and ERF feature selection methods, respectively). The rest of them presented a diminishing tendency that started from the early beginning.

The classifiers having the lowest performance were DT and SVM (see Figure 2a,b). The F1-score curves of the DT classifier had values slightly under 0.75. On the other hand, some of the SVM’s F1-scores reached 0.75.

When the Siena dataset and the FS1 were used, several classification models returned lower performance in comparison with the CHB-MIT experiments (see Figure 3a–c,e).

The best classification model was RF (see Figure 3d), and the combination RF-ERF reached an F1-score of 0.85. SVM and RR decreased faster than the rest of the FSMs.

The ANN model reached a performance of 0.80 for some combinations (ANN-LIME, ANN-SHAP). The RR curve decreased faster than the rest of the FSMs. SVM and the DT had a better performance than RR, but not as good as LIME and SHAP (see Figure 3c). The DT (see Figure 3a) showed a steadier behavior compared to all the classifiers, but its performance was around 0.70

Figure 3b depicts an interesting pattern. The F1-score was 0.5 at the beginning of the training, then several curves decreased almost from the beginning of the training (brown, blue, and purple lines). On the other hand, the LIME curve rose markedly as the number of features reduced.

When the CHB-MIT dataset and FS2 were used, the performance was better than the performance obtained during the FS1 experiments. Once again, the RR experiments tended to show poor performance and a faster decay in comparison to the rest of the FSMs.

The best performances were reported by the KNN model (see Figure 4e) and the worst by the DT and SVM models (see Figure 4a,b). KNN reported the best F1-score (0.90), and it corresponded to the experiments that removed a low number of features (e.g., 500 and 450). The combinations KNN-ERF-400/350/300/250/200 also reported an F1-score of 0.90.

The second-best-performing classifier was the ANN (see Figure 4c); most of the F1-score values were between 0.85 and 0.90. Most of the curves depicted a similar tendency, but the RR curve. RF (see Figure 4d) returned some F1-scores around 0.85, and these were slightly lower than the ANN scores.

When the Siena dataset and FS2 were used, the performances were a bit worse than the CHB-MIT experiments. The SVM classifier (see Figure 5b) depicted a similar pattern to the one observed in Figure 3b); when the number of features was reduced, the LIME and SHAP curves showed an increase in performance.

The best classification models were RF and KNN (see Figure 5d,e). These classifiers reported F1-scores around 0.85. For the KNN case, the RR curve (brown line) did not decay as fast as it did for the rest of the classifiers; however, RR was still the FSM with the worst performance.

Finally, to perform the comparison of the C-FSM combination and the feature selected, different cutoffs were selected. It is important to mention that based on the average of the F1-scores across every experiment, the decrease in classification performance between two consecutive cutoffs points was approximately equal, so based on the visual inspection of the Figure 2, Figure 3, Figure 4 and Figure 5, four cutoffs were defined: 450, 150, 100, and 50 features. Notice that we discarded analyzing cutoff points under 50 features, because several models showed an F1-score lower than 0.6.

### 3.2. Comparison of C-FSM Combinations

This analysis intended to observe, in detail, the performance of several C-FSM combinations. To do so, several cutoff points were chosen, then, to every cutoff point, the F1-score, sensitivity, and accuracy of all the combinations for a model classifier and feature selection method were computed. For the best-performing combinations of C-FSMs, the 5 × 2 CV F-test was applied to find statistically significant differences between the error rates.

Table 6, Table 7, Table 8 and Table 9 depict the different C-FSM tuples and their respective F1-score, sensitivity, and accuracy for different sizes of the feature vector. In particular, these tables show the performances when the 450, 150, 100, and 50 best features were kept.

When 450 features were used, it is depicted in Table 6 that there was not an FSM that outperformed the rest. The best performances were obtained by the KNN and ANN models, the former using the FS2 and the combination KNN-SVM/SHAP/ERF (0.90) and the latter using FS1; the best combination was ANN-SVM/SHAP/ERF (0.84). On the other hand, the F1-scores of the Siena dataset experiments were lower, on average. The combinations having the best performance were KNN-SVM/LIME (0.83) and KNN-DT/SVM/LIME/SHAP/ERF/RR (0.86), for FS1 and FS2, respectively.

Observe that the experiments using FS2 tended to report better performances than those in the FS1 experiments, no matter the dataset used. Furthermore, it should be noted that there were large differences between the accuracy and the F1-score values, due to the large class imbalance. The performances of the DT and SVM classifiers were noticeably lower than ANN/RF/KNN, the SVM being the classifier with the worst performance values.

Table 7 shows the classification metrics for the 150-feature experiments. The best performances, using the CHB-MIT dataset and FS1, were returned by the combination ANN-LIME/SHAP/ERF (0.85); for the FS2 case, this was KNN-SVM/ERF (0.89). On the other hand, the Siena experiments showed that the best-performing combinations included the ERF as an FSM; RF-ERF returned the largest F1-scores, and these were 0.85 and 0.86 for FS1 and FS2, respectively.

In the 100-feature case (see Table 8), the behavior was similar to the 450-feature case, in the sense that the best-suited FSM depended on the feature set and dataset. For the CHB-MIT dataset, the combinations with the best performances were KNN-SVM (0.85) and KNN-DT/SVM/ERF (0.87) for FS1 and FS2, respectively. For the Siena dataset, the best performance was reported by RF-ERF (0.85 and 0.86).

Table 9 shows that classifiers accounting for the best performances, RF and KNN, 0.84 and 0.85 being the largest values for both of the datasets. It is interesting to note that the best performances were similar for both datasets and the feature sets.

It is worth noticing that the RF classifier presented the most steady performance (≈0.8) no matter the FSM, dataset, and feature reduction (see Table 6, Table 7, Table 8 and Table 9).

In order to identify the significant differences between FSMs, Table 10 and Table 11 show the results of the F-test; given a dataset, a number of features, and a feature set (FS1 or FS2), the F-test was applied to the results of the 5 × 2 CV experiments. During the testing, the best-performing C-FSM (as per the F1-score) was compared to the rest of the FSMs. It must be considered that the 5 × 2 CV F-test evaluates the error rates, not the F1-scores, so the accuracy is computed and depicted in Table 6, Table 7, Table 8 and Table 9.

In the case of several combinations having the same F1-score, the sensitivity and accuracy were considered to choose the best-performing combinations.

The 450 feature section of Table 10 and Table 11 shows that most of the test results were not statistically significant (*p* > 0.05). In Table 10, there were three cases where the FSM error rates resulted in being statistically different from the rest of the FSMs, and these were RF-ERF-150 (Siena dataset), RF-ERF-100 (Siena dataset), and KNN-SVM-50 (CHB-MIT dataset). It must be noted that the differences in accuracy may be small (see Table 6, Table 7, Table 8 and Table 9), even if there are statistical differences.

In Table 11, there are no cases where an FSM was statistically different from the rest of the FSMs. It was observed that the RR experiments tended to show a statistical significance that did not depend on the number of features or the dataset.

Finally, Table 10 and Table 11 show that the most common combinations were KNN-SVM and RF-ERF.

### 3.3. Comparison of Selected Features

To determine if there were coincidences in the features selected by the FSMs and if the FSMs assigned more importance to the same features, the Jaccard index [64] was used to calculate the similarity by pairs of FSMs. For this analysis, the FS2 experiments were chosen because those experiments produced higher performances in comparison with FS1.

**Figure 6:**
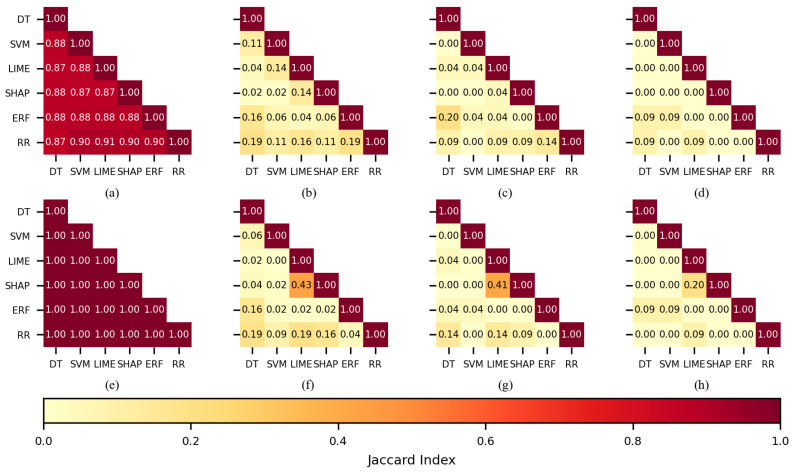
The similarity of the sets of features selected per each feature selection method when KNN was used as a classifier. CHB-MIT dataset: (**a**) best 450 features, (**b**) best 150, (**c**) best 100, and (**d**) best 50. Siena dataset: (**e**) best 450 features, (**f**) best 150, (**g**) best 100, and (**h**) best 50.

When 450 features were used for training the KNN classifier (see Figure 6a,e), the feature sets practically overlapped; indices had values over 0.85 for the CHB-MIT dataset and the Siena dataset. This behavior was expected, as it is highly probable to select similar features when the number of features in a dataset is large, so the 450 case will not be further discussed.

When 150 features were used during training (see Figure 6b,f), the Jaccard index for SHAP-LIME (0.43) was the largest of all the combinations when the Siena dataset was used; it was equivalent to 91 out of 150 features. For the CHB-MIT dataset, RR-ERF (0.19) and RR-DT (0.19) obtained the largest values. Figure 6c,d,g,h show a low coincidence for most of the FSMs when 100 or 50 features were used, and this applies to both datasets. The only notorious index was 0.41 belonging to SHAP-LIME (see Figure 6g).

**Figure 7:**
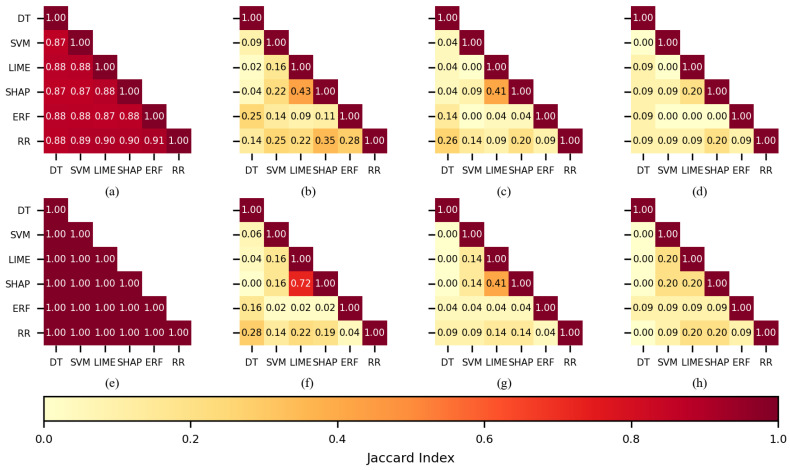
The similarity of the sets of features selected per each feature selection method when SVM was used as a classifier. CHB-MIT dataset: (**a**) best 450 features, (**b**) best 150, (**c**) best 100, and (**d**) best 50. Siena dataset: (**e**) best 450 features, (**f**) best 150, (**g**) best 100, and (**h**) best 50.

In the case of the SVM classifier, by using 150 features (see Figure 7b,f), the largest values were obtained by SHAP-LIME (0.43 and 0.72); the FSMs coincided in selecting 91 features for the CHB-MIT dataset and 126 for the Siena dataset. The Jaccard indices computed for 100 features (see Figure 7c,g) showed a good similarity for SHAP-LIME (0.41), and the selected feature sets coincided in 59 features for both of the datasets. When 50 features were used for training (see Figure 7d,h), several combinations returned a similarity value of 0.2.

**Figure 8:**
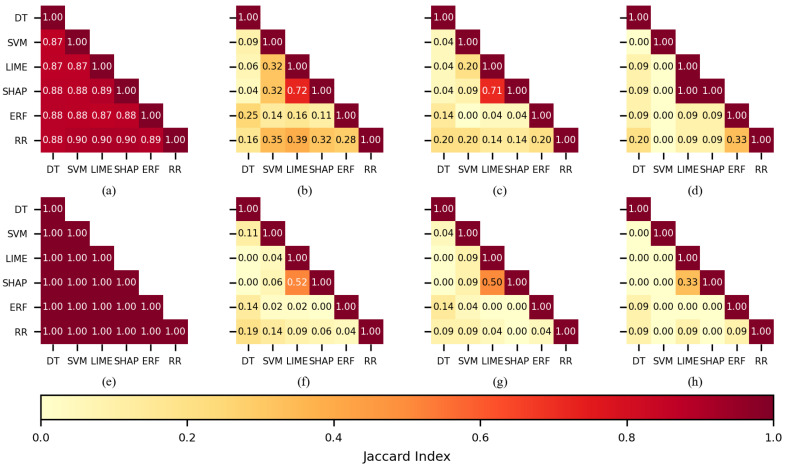
The similarity of the sets of features selected per each feature selection method when ANN was used as a classifier. CHB-MIT dataset: (**a**) best 450 features, (**b**) best 150, (**c**) best 100, and (**d**) best 50. Siena dataset: (**e**) best 450 features, (**f**) best 150, (**g**) best 100, and (**h**) best 50.

The ANN classifier followed a similar pattern as SVM; SHAP-LIME obtained larger indices than most of the combinations, and these were 0.72 and 0.52 for 150 features, 0.71 and 0.50 for 100, and 1.0 and 0.33 for 50 (see Figure 8b–d,f–h), respectively. It should be noted that a Jaccard index of 1 means that both sets totally overlapped.

**Figure 9:**
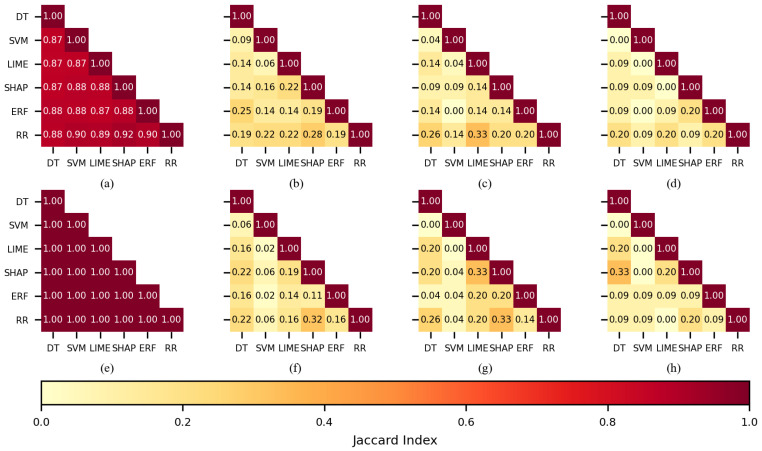
The similarity of the sets of features selected per each feature selection method when DT was used as a classifier. CHB-MIT dataset: (**a**) best 450 features, (**b**) best 150, (**c**) best 100, and (**d**) best 50. Siena dataset: (**e**) best 450 features, (**f**) best 150, (**g**) best 100, and (**h**) best 50.

Interestingly, when the DT classifier was used, the SHAP-LIME similarity was equal to or lower than other combinations. When using 150 features, the largest indices were returned by the combination RR-SHAP (see Figure 9b,f). Figure 9c,g,h show that the largest similarity values were returned by combinations including LIME or SHAP.

**Figure 10:**
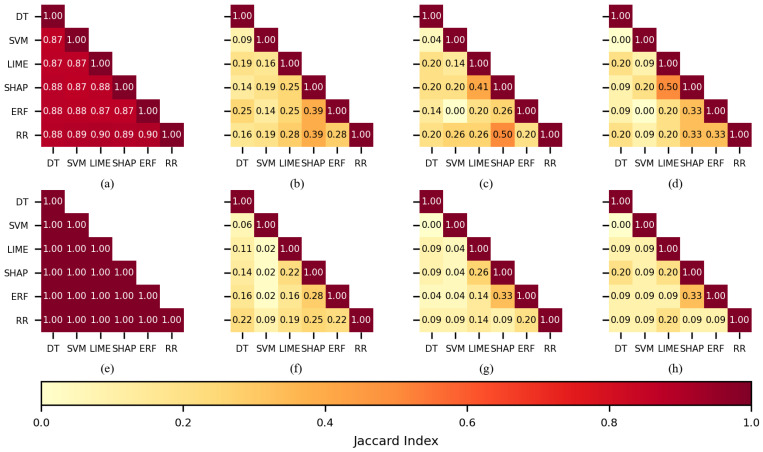
The similarity of the sets of features selected per each feature selection method when RF was used as a classifier. CHB-MIT dataset: (**a**) best 450 features, (**b**) best 150, (**c**) best 100, and (**d**) best 50. Siena dataset: (**e**) best 450 features, (**f**) best 150, (**g**) best 100, and (**h**) best 50.

Comparable to the DT, the RF case showed the largest values when a combination included LIME or SHAP. A remarkable fact is that there were two combinations having an index value of 0.5 (see Figure 10c,d).

**Figure 11:**
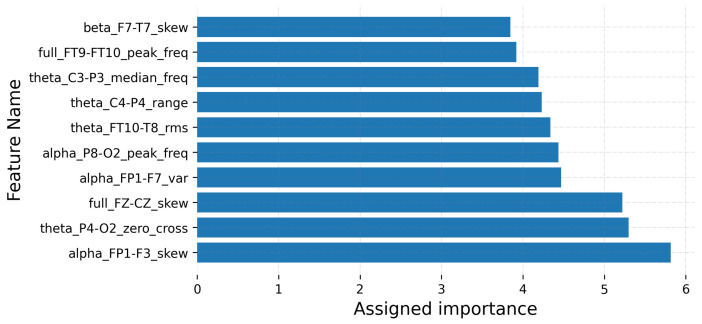
Features having the largest assigned importance. The results correspond to the combination KNN-SVM and the CHB-MIT dataset. Importance values are not normalized.

**Figure 12:**
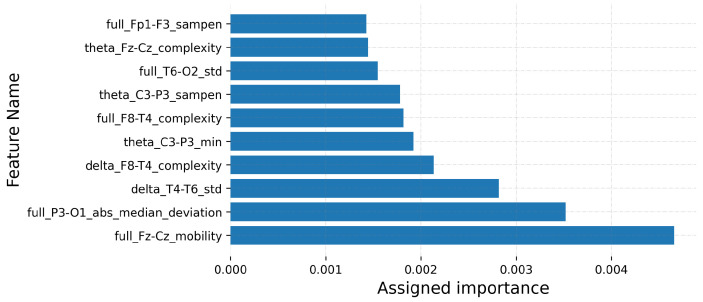
Features having the largest assigned importance. The results correspond to the combination RF-ERF and the Siena dataset. Importance values are not normalized.

In order to compare the selected features for the best C-FSM combinations in Table 9, Figure 11 and Figure 12 show the top-10 features for KNN-SVM and RF-ERF, respectively. The former figure corresponds to the CHB-MIT dataset, while the latter to the Siena dataset. Each feature is defined as follows: EEG band/bipolar channel/metric. For example, in Figure 11, the feature with the greatest importance value is “alpha_FP1-F3_skew”, which indicates that the most important feature was the skewness measured in the bipolar channel FP1-F3 on the alpha band. It is important to note that the selected features may vary due to several factors, including seizure type, epileptogenic region, and EEG montage, among others.

## 4. Discussion

In the evaluation of feature dimensionality reduction analysis, it was observed that the best machine learning classifiers were ANN, RF, and KNN, taking into account neither the database nor the FS. This is evident by looking at the performance tendency of every combination of C-FSMs (colored lines). It is important to note that there was a recurrent behavior in all the combinations, that is almost all of them started showing a performance decrease when the feature vectors were around a length of 50. Lower than that, the scores began to be around 0.6 or less. Therefore, a feature vector of a length of 50 is the minimum suggested for having good classification performance using some of the C-FSM combinations, for FS in epilepsy databases. Moreover, if FS2 is used, the scores achieved by most of the C-FSM combinations were improved and steadier. In this sense, the DT and RF classifiers were less affected by the dimensionality reduction no matter the database, the FS, nor the FSM. This could be explained by the nature of those algorithms, that is both algorithms use a ranking metric to determine the importance of each feature vector in a particular classification task.

Regarding to the comparison of C-FSM combinations, it can be noticed that the models that were trained with CHB-MIT data had better F1-scores than the ones trained with Siena data. We considered that the performance difference was due to the CHB-MIT database being larger than the Siena Scalp EEG database. Actually, the number of CHB-MIT epochs used during experimentation was more than twice the number of Siena epochs. Another aspect to consider is the number of channels, 21 and 18 for the CHB-MIT and Siena datasets, respectively.

In addition, we can observe in Table 6, Table 7, Table 8 and Table 9 that the greater the number of features, the ANN and KNN showed better F1-scores; on the other hand, the lower the number of features, KNN kept showing the best F1-scores, and RF emerged with better F1-scores than the ANN. Note that, even though KNN presented the highest F1-scores, the difference between KNN and RF reduced as the number of features decreased.

Furthermore, in Table 10 and Table 11, it can be seen that the smaller the number of features, the greater the number of significant differences was between the best C-FSM and the other FSMs. In this sense, these significant differences in the classifiers’ performance indicate a relationship between the classifier and the feature selection method; in this case, for a lower number of features, KNN was better when using SVM and ERF, while RF was better when using ERF.

The main purpose of this study was not to train a classifier for seizure classification from EEG data; however, it can be considered that the performances obtained, in particular for KNN and RF, were similar to other studies when experiments were conducted under similar conditions, specifically the same classifier and EEG database.

In this sense, comparing our work with the state-of-the-art, in [12], the authors used the CHB-MIT database, seven feature selection methods, nine classifiers, and selecting the top 20 or 25 features, obtaining the best mean classification error of 0.12 by using the KNN classifier. Nonetheless, the authors tested their methodology neither using a wider spectrum of feature vector sizes, nor using different databases. In this work, we found that having the top 50 features, on the same database, the combinations KNN-SVM-50 (FS1), KNN-SVM-50 (FS2), and KNN-ERF-50 (FS2) (see Table 9) returned a mean classification error of 0.03. Moreover, the results in [12] correspond to a balanced dataset, while in this work, the proportion between seizure and no-seizure was 1:9, respectively, our methodology being more appropriate for seizure and no-seizure detection considering that epilepsy datasets are naturally unbalanced.

Additionally, Kathi and Ingle [25] presented an accuracy of 0.97 and an F1-score of 0.97 by using KNN and 11 feature metrics, testing their methodology on the Bonn University database [65] and using an equal proportion of healthy and seizure instances for the training and test sets. Furthermore, they reported a reduction in the feature set used to compute the feature vectors instead of directly reducing the size of the feature vectors. Hence, the authors did not evaluate either different sizes of the feature vectors or the different feature selection methods. On the contrary, in the present work, several experiments reported an accuracy of 0.97, but had a lower F1-score (0.84 and 0.85); however, these lower scores were expected considering that, for unbalanced datasets, the F1-score is more reliable.

On the other hand, RR presented the worst results no matter the classifier used, contrasting with [54], who reported RR as one of the FSMs that performed better and also provided good stability across datasets. However, the nature of the datasets used in [54] was different from EEG, which indicates that RR is not appropriate to be used for seizure and no-seizure detection.

Finally, concerning the comparison of selected features, by using the Jaccard index, it was observed that most of the time, SHAP-LIME returned the largest levels of similarity, meaning that both FMSs usually selected the same features, even though they did not present the best performance on the classification; on the contrary, SVM and ERF, which were the best FSMs for KNN and RF, respectively, did not present a higher Jaccard index.

Large values in SHAP-LIME were partially expected because, as explained in [49,50], SHAP and LIME are designed to be model-agnostic and to explain the classification model. In addition, the SHAP implementation applied in this study (kernel SHAP) followed a similar approach to LIME, but different functions were applied for the estimation of the locality and the similarity between the classification *f* and interpretable *g* model. In the case of SVM and ERF, the low levels of the similarity of features selected were partially explained because the nature and training procedure of SVM are quite different from tree-based models.

Therefore, this experiment showed that two different feature spaces might result in a good classification performance, such as the features selected for KNN-SVM and RF-ERF (Figure 11 and Figure 12). On the contrary, the RR method presented good similarity values compared to other FSMs; nonetheless, it presented the worst classification performances.

## 5. Conclusions and Future Work

In the present study, six FSMs were compared to define, first, the minimum number of features that can be chosen for each FSM without sacrificing the classifiers’ performances, second, the performance of several C-FSM combinations to discover if a relationship exists between the FSMs with a particular classification algorithm, and third, if there is a feature or feature set that remains across different C-FSM combinations.

We can conclude that when the number of selected features was drastically reduced (100 and 50 features), the differences between classifiers’ performance increased, but none of the FSMs showed a predominance over the rest. Furthermore, it was observed that it was possible to perform a large reduction of the number of features while having a low impact on the model performance until having a 50-feature vector length.

The results indicated that the classifiers’ performance might be affected by diverse factors such as the EEG database, the features, and the number of features. However, the combinations KNN-SVM and RF-ERF are advised. Furthermore, the use of RR is not appropriate for seizure EEG data, as it yielded lower performances than the rest of the FSMs, and it was more time-consuming.

Regarding the proposed feature sets, FS2 is suggested to be used on seizure and no-seizure classification problems, given that the performance of several C-FSMs was improved while using it.

In future work, some opportunity areas could be explored: First, a more extended analysis is required to evaluate the FSMs in combination with deep learning models, including a more extensive parameter tuning process and the use of more complex features. Furthermore, considering that our results showed that RF-ERF obtained one of the best performances, it would be interesting to perform an evaluation of tree-based models for feature selection and classification of seizure and no-seizure EEG data.

Second, FSM stability was not evaluated in this study; the evaluation of stability with instance perturbation, as proposed by [48], would help to evaluate the robustness of an FSM against small variations in the EEG dataset, because variations can be caused by inter-subject variability or noise.

Finally, assessing a subject cross-validation methodology would be interesting for future work to test inter-subject performances.

## Figures and Tables

**Table 1:** Features’ set.

Feature Set 1 (FS1)	Feature Set 2 (FS2)
Median Frequency (i) [39]	Minimum (ix) [34]
Variance (ii) [40]	Complexity (x) [34]
Skewness (iii) [40]	Mobility (xi) [34]
Kurtosis (iv) [40]	Interquartile Range (xii) [40]
Peak Frequency (v) [41]	Median Absolute Deviation (xiii) [42]
Root Mean Square (vi) [40]	Sample Entropy (xiv) [43]
Range (vii) [40]	Mean (xv) [40]
Number of Zero Crossings (viii) [34]	Standard Deviation (xvi) [40]

**Table 2:** Structure of ANN layers.

Layer	Type	Size	Activation
1	Dense	100	ReLU
2	Dense	50	ReLU
3	Dense	1	Sigmoid

**Table 3:** Configuration setting per classification method. It was adjusted from FS1.

Method	Parameter	Selected Value	Parameters Grid
DT	Min. samples per node	8	2, 4, 8, 16
	Split criterion	Gini impurity	Gini impurity
SVM	Regularization parameter	2	0.1, 0.5, 1, 2, 10
	Kernel	RBF	Linear, RBF
	Gamma coefficient	1/(n_features×var(x))	1/(n_features×var(x)), 1/n_features
KNN	Num. of neighbors	15	3, 5, 10, 15
	Distance metric	Manhattan	Euclidean, Manhattan
	Weight function	Uniform	Uniform, distance-based
RF	Num. of trees	150	30, 50, 100, 150
	Min. samples per node	4	2, 4, 8, 16
ANN	Optimizer	Adam	
	Loss measure	Cross-entropy	
	Epochs	200	

**Table 4:** Configuration setting per classification method. It was adjusted from FS2.

Method	Parameter	Selected Value
DT	Min. samples per node	2
	Split criterion	Gini impurity
SVM	Regularization parameter	2
	Kernel	RBF
	Gamma coefficient	1/(n_features×var(x))
KNN	Num. of neighbors	10
	Distance metric	Euclidean
	Weight function	Distance
RF	Num. of trees	150
	Min. samples per node	4
ANN	Optimizer	Adam
	Loss measure	Cross-entropy
	Epochs	200

**Table 5:** Configuration setting per feature selection method.

Method	Parameter	Values
DT	Min. samples per node	4
	Split criterion	Gini impurity
SVM	Regularization parameter *C*	1
	Kernel	Linear kernel
LIME	Discretization of values	No
SHAP	Length of background dataset	50
ERF	Num. of trees	10
	Min. samples per node	2
	Split criterion	Gini impurity

**Table 6:** Performance comparison using 450 features. The largest F1-scores are displayed in **red bold** and **blue bold** for FS1 and FS2, respectively. F1s = F1-score, Se = sensitivity, Acc = accuracy.

		DT			SVM			ANN			RF			KNN		
CHB-MIT dataset																
FSM		F1s	Se	Acc	F1s	Se	Acc	F1s	Se	Acc	F1s	Se	Acc	F1s	Se	Acc
DT	FS1	0.72	0.73	0.94	0.69	0.53	0.95	0.82	0.76	0.96	0.81	0.69	0.96	0.78	0.65	0.96
	FS2	0.74	0.74	0.94	0.75	0.61	0.95	0.86	0.81	0.97	0.84	0.73	0.97	0.89	0.83	0.98
SVM	FS1	0.72	0.73	0.94	0.70	0.55	0.95	**0.84**	0.78	0.97	0.81	0.69	0.96	0.82	0.72	0.96
	FS2	0.73	0.74	0.94	0.76	0.63	0.96	0.86	0.80	0.97	0.84	0.73	0.97	**0.90**	0.84	0.98
LIME	FS1	0.72	0.72	0.94	0.72	0.58	0.95	0.83	0.77	0.96	0.80	0.68	0.96	0.78	0.65	0.96
	FS2	0.73	0.74	0.94	0.76	0.63	0.96	0.87	0.81	0.97	0.84	0.73	0.97	0.89	0.83	0.98
SHAP	FS1	0.72	0.73	0.94	0.70	0.56	0.95	**0.84**	0.77	0.97	0.81	0.69	0.96	0.78	0.65	0.96
	FS2	0.73	0.74	0.94	0.76	0.62	0.96	0.87	0.82	0.97	0.84	0.73	0.97	**0.90**	0.83	0.98
ERF	FS1	0.72	0.73	0.94	0.70	0.55	0.95	**0.84**	0.78	0.97	0.82	0.70	0.96	0.80	0.68	0.96
	FS2	0.74	0.74	0.94	0.75	0.61	0.95	0.86	0.81	0.97	0.84	0.73	0.97	**0.90**	0.84	0.98
RR	FS1	0.67	0.68	0.93	0.62	0.46	0.94	0.73	0.63	0.95	0.73	0.59	0.95	0.73	0.59	0.95
	FS2	0.71	0.72	0.94	0.71	0.56	0.95	0.84	0.78	0.97	0.81	0.69	0.96	0.89	0.82	0.97
**Siena Scalp EEG dataset**																
**FSM**		**F1s**	**Se**	**Acc**	**F1s**	**Se**	**Acc**	**F1s**	**Se**	**Acc**	**F1s**	**Se**	**Acc**	**F1s**	**Se**	**Acc**
DT	FS1	0.69	0.69	0.91	0.48	0.33	0.90	0.75	0.69	0.93	0.80	0.68	0.95	0.82	0.77	0.95
	FS2	0.71	0.71	0.91	0.53	0.37	0.90	0.78	0.71	0.94	0.85	0.75	0.96	**0.86**	0.84	0.96
SVM	FS1	0.69	0.69	0.91	0.52	0.36	0.90	0.75	0.68	0.93	0.80	0.68	0.95	**0.83**	0.79	0.95
	FS2	0.71	0.72	0.91	0.53	0.37	0.90	0.78	0.74	0.94	0.85	0.75	0.96	**0.86**	0.84	0.96
LIME	FS1	0.69	0.70	0.91	0.54	0.38	0.91	0.78	0.72	0.94	0.81	0.69	0.95	**0.83**	0.79	0.95
	FS2	0.71	0.71	0.91	0.53	0.37	0.90	0.77	0.73	0.93	0.85	0.76	0.96	**0.86**	0.84	0.96
SHAP	FS1	0.69	0.70	0.91	0.51	0.35	0.90	0.77	0.71	0.94	0.81	0.70	0.95	0.82	0.79	0.95
	FS2	0.71	0.71	0.91	0.53	0.37	0.90	0.78	0.71	0.94	0.85	0.75	0.96	**0.86**	0.84	0.96
ERF	FS1	0.70	0.70	0.91	0.47	0.32	0.90	0.75	0.68	0.93	0.82	0.71	0.95	0.67	0.68	0.95
	FS2	0.71	0.72	0.91	0.53	0.36	0.90	0.79	0.75	0.94	0.85	0.76	0.96	**0.86**	0.84	0.96
RR	FS1	0.67	0.68	0.90	0.28	0.17	0.88	0.68	0.60	0.92	0.76	0.62	0.94	0.78	0.72	0.94
	FS2	0.70	0.71	0.91	0.51	0.35	0.90	0.78	0.73	0.94	0.85	0.75	0.96	**0.86**	0.83	0.96

**Table 7:** Performance comparison using 150 features. The largest F1-scores are displayed in **red bold** and **blue bold** for FS1 and FS2, respectively. F1s = F1-score, Se = sensitivity, Acc = accuracy.

		DT			SVM			ANN			RF			KNN		
CHB-MIT dataset																
FSM		F1s	Se	Acc	F1s	Se	Acc	F1s	Se	Acc	F1s	Se	Acc	F1s	Se	Acc
DT	FS1	0.73	0.74	0.94	0.68	0.53	0.94	0.83	0.77	0.96	0.82	0.71	0.96	0.70	0.55	0.95
	FS2	0.74	0.74	0.94	0.74	0.60	0.95	0.85	0.79	0.97	0.84	0.74	0.97	0.87	0.79	0.97
SVM	FS1	0.73	0.73	0.94	0.66	0.50	0.94	0.84	0.77	0.97	0.82	0.71	0.96	0.82	0.73	0.96
	FS2	0.74	0.75	0.94	0.72	0.58	0.95	0.86	0.79	0.97	0.84	0.74	0.97	**0.89**	0.82	0.97
LIME	FS1	0.72	0.73	0.94	0.75	0.62	0.95	**0.85**	0.80	0.97	0.81	0.69	0.96	0.69	0.54	0.95
	FS2	0.73	0.74	0.94	0.80	0.68	0.96	0.87	0.82	0.97	0.84	0.74	0.97	0.86	0.77	0.97
SHAP	FS1	0.73	0.74	0.94	0.73	0.59	0.95	**0.85**	0.80	0.97	0.80	0.68	0.96	0.71	0.56	0.95
	FS2	0.74	0.75	0.94	0.76	0.63	0.96	0.87	0.82	0.97	0.83	0.73	0.97	0.86	0.77	0.97
ERF	FS1	0.73	0.74	0.94	0.74	0.60	0.95	**0.85**	0.79	0.97	0.83	0.72	0.97	0.80	0.69	0.96
	FS2	0.74	0.75	0.94	0.72	0.58	0.95	0.86	0.80	0.97	0.84	0.74	0.97	**0.89**	0.83	0.98
RR	FS1	0.63	0.63	0.92	0.51	0.35	0.93	0.63	0.51	0.93	0.66	0.50	0.94	0.57	0.41	0.93
	FS2	0.67	0.68	0.93	0.59	0.42	0.93	0.77	0.68	0.95	0.76	0.62	0.96	0.84	0.74	0.97
**Siena Scalp EEG dataset**																
**FSM**		**F1s**	**Se**	**Acc**	**F1s**	**Se**	**Acc**	**F1s**	**Se**	**Acc**	**F1s**	**Se**	**Acc**	**F1s**	**Se**	**Acc**
DT	FS1	0.70	0.70	0.91	0.30	0.18	0.88	0.73	0.66	0.93	0.82	0.71	0.95	0.72	0.64	0.93
	FS2	0.72	0.73	0.91	0.41	0.26	0.89	0.74	0.66	0.93	0.85	0.76	0.96	0.85	0.82	0.95
SVM	FS1	0.66	0.67	0.90	0.42	0.27	0.89	0.71	0.64	0.93	0.77	0.63	0.94	0.80	0.75	0.94
	FS2	0.70	0.71	0.91	0.61	0.45	0.91	0.78	0.72	0.94	0.83	0.73	0.95	0.85	0.81	0.95
LIME	FS1	0.69	0.70	0.91	0.62	0.47	0.92	0.80	0.76	0.94	0.82	0.71	0.95	0.80	0.74	0.94
	FS2	0.71	0.71	0.91	0.69	0.55	0.92	0.83	0.80	0.95	0.84	0.74	0.96	0.85	0.81	0.95
SHAP	FS1	0.71	0.71	0.91	0.56	0.40	0.91	0.79	0.75	0.94	0.83	0.73	0.95	0.80	0.75	0.94
	FS2	0.71	0.72	0.91	0.67	0.52	0.92	0.82	0.78	0.95	0.85	0.77	0.96	0.85	0.81	0.95
ERF	FS1	0.71	0.72	0.92	0.30	0.18	0.88	0.75	0.71	0.93	**0.85**	0.76	0.96	0.64	0.65	0.93
	FS2	0.72	0.73	0.92	0.40	0.25	0.89	0.76	0.71	0.93	**0.86**	0.77	0.96	0.83	0.78	0.95
RR	FS1	0.64	0.65	0.90	0.16	0.09	0.87	0.57	0.47	0.90	0.66	0.50	0.93	0.66	0.56	0.92
	FS2	0.67	0.68	0.90	0.29	0.17	0.87	0.62	0.52	0.91	0.78	0.65	0.94	0.81	0.76	0.94

**Table 8:** Performance comparison using 100 features. The largest F1-scores are displayed in **red bold** and **blue bold** for FS1 and FS2, respectively. F1s = F1-score, Se = sensitivity, Acc = accuracy.

		DT			SVM			ANN			RF			KNN		
CHB-MIT dataset																
FSM		F1s	Se	Acc	F1s	Se	Acc	F1s	Se	Acc	F1s	Se	Acc	F1s	Se	Acc
DT	FS1	0.73	0.74	0.94	0.68	0.53	0.95	0.81	0.74	0.96	0.83	0.72	0.97	0.73	0.60	0.95
	FS2	0.74	0.75	0.94	0.72	0.58	0.95	0.84	0.78	0.97	0.84	0.74	0.97	**0.87**	0.79	0.97
SVM	FS1	0.73	0.73	0.94	0.67	0.52	0.94	0.83	0.76	0.96	0.83	0.72	0.97	**0.85**	0.76	0.97
	FS2	0.74	0.75	0.94	0.68	0.52	0.94	0.84	0.77	0.97	0.84	0.74	0.97	**0.87**	0.80	0.97
LIME	FS1	0.72	0.73	0.94	0.74	0.61	0.95	0.84	0.77	0.96	0.81	0.69	0.96	0.65	0.49	0.94
	FS2	0.73	0.74	0.94	0.79	0.68	0.96	0.86	0.80	0.97	0.84	0.74	0.97	0.83	0.73	0.96
SHAP	FS1	0.74	0.74	0.94	0.73	0.59	0.95	0.84	0.78	0.97	0.80	0.68	0.96	0.68	0.52	0.94
	FS2	0.74	0.75	0.94	0.75	0.63	0.95	0.85	0.80	0.97	0.83	0.72	0.97	0.83	0.73	0.97
ERF	FS1	0.74	0.74	0.94	0.70	0.55	0.95	0.83	0.75	0.96	0.83	0.73	0.97	0.82	0.72	0.96
	FS2	0.74	0.75	0.94	0.71	0.56	0.95	0.85	0.79	0.97	0.85	0.74	0.97	**0.87**	0.80	0.97
RR	FS1	0.61	0.62	0.91	0.44	0.29	0.92	0.59	0.46	0.93	0.65	0.49	0.94	0.46	0.30	0.92
	FS2	0.66	0.67	0.93	0.54	0.37	0.93	0.74	0.65	0.95	0.73	0.58	0.95	0.80	0.68	0.96
**Siena Scalp EEG dataset**																
**FSM**		**F1s**	**Se**	**Acc**	**F1s**	**Se**	**Acc**	**F1s**	**Se**	**Acc**	**F1s**	**Se**	**Acc**	**F1s**	**Se**	**Acc**
DT	FS1	0.71	0.72	0.91	0.24	0.14	0.87	0.70	0.63	0.92	0.83	0.73	0.96	0.70	0.61	0.92
	FS2	0.72	0.72	0.91	0.36	0.22	0.88	0.71	0.63	0.92	0.85	0.76	0.96	0.83	0.80	0.95
SVM	FS1	0.66	0.67	0.90	0.35	0.22	0.89	0.70	0.63	0.92	0.78	0.66	0.95	0.75	0.69	0.93
	FS2	0.69	0.70	0.91	0.61	0.46	0.91	0.78	0.73	0.94	0.82	0.72	0.95	0.84	0.81	0.95
LIME	FS1	0.69	0.70	0.91	0.64	0.50	0.92	0.77	0.72	0.94	0.81	0.71	0.95	0.78	0.71	0.94
	FS2	0.71	0.72	0.91	0.71	0.58	0.93	0.82	0.78	0.95	0.84	0.75	0.96	0.84	0.80	0.95
SHAP	FS1	0.70	0.71	0.91	0.55	0.40	0.91	0.78	0.74	0.94	0.84	0.74	0.96	0.78	0.73	0.94
	FS2	0.71	0.72	0.91	0.70	0.57	0.93	0.81	0.77	0.94	0.85	0.77	0.96	0.83	0.80	0.95
ERF	FS1	0.72	0.72	0.92	0.32	0.20	0.88	0.74	0.67	0.93	**0.85**	0.76	0.96	0.62	0.63	0.92
	FS2	0.73	0.74	0.92	0.28	0.16	0.87	0.72	0.65	0.92	**0.86**	0.77	0.96	0.80	0.73	0.94
RR	FS1	0.62	0.63	0.89	0.14	0.07	0.87	0.51	0.39	0.89	0.64	0.47	0.92	0.61	0.49	0.91
	FS2	0.65	0.66	0.89	0.25	0.14	0.87	0.58	0.47	0.90	0.77	0.64	0.94	0.76	0.69	0.93

**Table 9:** Performance comparison using 50 features. The largest F1-scores are displayed in **red bold** and **blue bold** for FS1 and FS2, respectively. F1s = F1-score, Se = sensitivity, Acc = accuracy.

		DT			SVM			ANN			RF			KNN		
CHB-MIT dataset																
FSM		F1s	Se	Acc	F1s	Se	Acc	F1s	Se	Acc	F1s	Se	Acc	F1s	Se	Acc
DT	FS1	0.73	0.74	0.94	0.68	0.53	0.94	0.79	0.70	0.96	0.83	0.72	0.97	0.70	0.56	0.95
	FS2	0.74	0.74	0.94	0.70	0.55	0.95	0.82	0.75	0.96	**0.84**	0.74	0.97	0.82	0.72	0.96
SVM	FS1	0.71	0.72	0.94	0.65	0.49	0.94	0.79	0.71	0.96	0.81	0.70	0.96	**0.85**	0.77	0.97
	FS2	0.73	0.74	0.94	0.63	0.47	0.94	0.81	0.73	0.96	0.83	0.73	0.97	**0.84**	0.75	0.97
LIME	FS1	0.72	0.73	0.94	0.73	0.59	0.95	0.81	0.73	0.96	0.78	0.65	0.96	0.60	0.44	0.94
	FS2	0.73	0.74	0.94	0.76	0.63	0.95	0.81	0.74	0.96	0.83	0.72	0.97	0.76	0.64	0.95
SHAP	FS1	0.74	0.74	0.94	0.73	0.59	0.95	0.80	0.73	0.96	0.78	0.65	0.96	0.62	0.46	0.94
	FS2	0.74	0.75	0.94	0.71	0.57	0.95	0.79	0.71	0.96	0.82	0.72	0.96	0.78	0.66	0.96
ERF	FS1	0.73	0.74	0.94	0.60	0.44	0.94	0.79	0.71	0.96	0.83	0.72	0.96	0.82	0.73	0.96
	FS2	0.74	0.75	0.94	0.65	0.49	0.94	0.82	0.74	0.96	**0.84**	0.74	0.97	**0.84**	0.75	0.97
RR	FS1	0.55	0.57	0.90	0.37	0.23	0.91	0.53	0.39	0.93	0.53	0.37	0.93	0.37	0.23	0.91
	FS2	0.64	0.65	0.92	0.48	0.32	0.92	0.64	0.52	0.94	0.69	0.54	0.95	0.70	0.55	0.95
**Siena Scalp EEG dataset**																
**FSM**		**F1s**	**Se**	**Acc**	**F1s**	**Se**	**Acc**	**F1s**	**Se**	**Acc**	**F1s**	**Se**	**Acc**	**F1s**	**Se**	**Acc**
DT	FS1	0.71	0.71	0.91	0.20	0.11	0.87	0.65	0.56	0.91	0.83	0.73	0.95	0.57	0.47	0.90
	FS2	0.72	0.73	0.92	0.21	0.12	0.87	0.64	0.53	0.91	0.84	0.75	0.96	0.79	0.73	0.94
SVM	FS1	0.63	0.64	0.89	0.28	0.16	0.88	0.64	0.54	0.91	0.75	0.61	0.94	0.66	0.56	0.92
	FS2	0.68	0.68	0.90	0.52	0.36	0.90	0.72	0.66	0.92	0.80	0.69	0.95	0.80	0.74	0.94
LIME	FS1	0.69	0.70	0.91	0.64	0.50	0.92	0.71	0.64	0.92	0.81	0.71	0.95	0.74	0.66	0.93
	FS2	0.70	0.71	0.91	0.67	0.53	0.92	0.75	0.69	0.93	0.83	0.73	0.95	0.81	0.76	0.94
SHAP	FS1	0.71	0.73	0.92	0.56	0.40	0.91	0.73	0.68	0.93	0.83	0.73	0.95	0.73	0.64	0.93
	FS2	0.71	0.72	0.91	0.68	0.55	0.92	0.77	0.71	0.93	0.84	0.76	0.96	0.82	0.77	0.95
ERF	FS1	0.72	0.72	0.92	0.21	0.11	0.87	0.64	0.55	0.91	**0.84**	0.75	0.96	0.63	0.64	0.89
	FS2	0.73	0.73	0.92	0.19	0.10	0.87	0.66	0.55	0.91	**0.85**	0.76	0.96	0.71	0.64	0.92
RR	FS1	0.63	0.64	0.89	0.14	0.07	0.87	0.40	0.27	0.88	0.59	0.43	0.91	0.49	0.36	0.89
	FS2	0.63	0.64	0.89	0.19	0.10	0.87	0.48	0.35	0.89	0.73	0.59	0.93	0.71	0.62	0.92

**Table 10:** Comparison of the best C-FSM when FS1 is used. It is compared against the rest of the FSMs. **•** denotes *p* < 0.05.

Best FSM	Dataset	Classifier	DT	SVM	LIME	SHAP	ERF	RR
450 features								
SVM	CHB-MIT	ANN						•
ERF	CHB-MIT	ANN						•
SVM	Siena	KNN						•
LIME	Siena	KNN	•					•
150 features								
LIME	CHB-MIT	ANN	•					•
SHAP	CHB-MIT	ANN	•					•
ERF	Siena	RF	•	•	•	•		•
100 features								
SVM	CHB-MIT	KNN	•		•	•		
ERF	Siena	RF	•	•	•	•		•
50 features								
SVM	CHB-MIT	KNN	•		•	•	•	•
ERF	Siena	RF		•	•			•

**Table 11:** Comparison of the best C-FSM when FS2 is used. It is compared against the rest of the FSMs. **•** denotes *p* < 0.05.

Best FSM	Dataset	Classifier	DT	SVM	LIME	SHAP	ERF	RR
450 features								
SVM	CHB-MIT	KNN			•	•		•
ERF	CHB-MIT	KNN	•		•	•		•
DT	Siena	KNN						•
SVM	Siena	KNN					•	•
LIME	Siena	KNN						•
SHAP	Siena	KNN						
ERF	Siena	KNN		•				
150 features								
ERF	CHB-MIT	KNN	•		•	•		•
ERF	Siena	RF		•	•			
100 features								
SVM	CHB-MIT	KNN			•	•		•
ERF	CHB-MIT	KNN			•	•		•
ERF	Siena	RF		•	•			•
50 features								
SVM	CHB-MIT	KNN	•		•	•		•
ERF	CHB-MIT	KNN	•		•	•		•
ERF	Siena	RF		•	•			•

## Data Availability

Publicly available datasets were analyzed in this study. The CHB-MIT Scalp EEG Database is available at https://physionet.org/content/chbmit/1.0.0/ (accessed on 18 October 2021) and the Siena Scalp EEG Database is available at https://physionet.org/content/siena-scalp-eeg/1.0.0/ (accessed on 18 October 2021).
